# Two putative Enterococcus faecalis fabG genes do not encode β-ketoacyl-acyl carrier protein reductases

**DOI:** 10.1099/mic.0.001610

**Published:** 2025-09-24

**Authors:** Qi Zou, Huijuan Dong, John E. Cronan

**Affiliations:** 1 Department of Microbiology, University of Illinois at Urbana-Champaign, Urbana, IL, USA; 2 Department of Biochemistry, University of Illinois at Urbana-Champaign, Urbana, IL, USA

**Keywords:** β-ketoacyl-acyl carrier protein (ACP) reductases, *Enterococcus faecalis*, FabG, fatty acid synthesis

## Abstract

*Enterococcus faecalis* encodes three putative *Escherichia coli* β-ketoacyl-acyl carrier protein (ACP) reductases (FabG). The *fabG1* gene is located within the operon that encodes most of the fatty acid synthesis genes, while the putative *fabG2* and *fabG3* genes are located elsewhere on the chromosome. The genes were tested for the ability to complement the growth of an *E. coli fabG*(Ts) strain at the non-permissive temperature. Of the three genes, only *E. faecalis* FabG1 restored growth at high temperature. Moreover, deletion of the *E. faecalis fabG1* gene resulted in an auxotrophic strain that required oleic acid for growth, arguing that it encodes the only functional β-ketoacyl-ACP reductase. Growth of this strain in the absence of fatty acid was restored by plasmid-borne *fabG1*, but not by plasmids encoding either *fabG2* or *fabG3*. Although *E. faecalis fabG2* has a putative binding site for the FabT transcription factor at the 5′ end of the coding region, expression of a transcriptional fusion with β-galactosidase was unaffected by deletion of *fabT* or by fatty acid supplementation.

**Impact Statement**

Fatty acid synthesis is essential for the assembly and function of the lipid bilayer of the *Enterococcus faecalis* cell membrane. The enzymes of the pathway are largely encoded in an 11-gene operon containing the *fabG* gene. Others have reported a second *fabG* gene, and a putative third *fabG* gene has been annotated. However, FabG is a member of the very large protein family, the short-chain reductase/dehydrogenase (SDR) family. These enzymes catalyse diverse reactions, often in monosaccharide pathways, thus giving functional annotations a degree of uncertainty. We report that only the *fabG* gene of the operon supports the growth of *fabG* mutant strains of *Escherichia coli* and *E. faecalis,* indicating the difficulties of annotation within the SDR family.

## Data Summary

Both of the two putative *fabG* genes are unable to replace the *in vivo* function of the *fabG* genes of *Escherichia coli* and *Enterococcus faecalis*. Deletion of the *fabG* gene located in the fatty acid synthesis operon results in fatty acid auxotrophy. The genome sequence of the *E. faecalis* strain studied (strain FA2-2) is Accession CP085841.1 in the NCBI GenBank nucleotide database. The data that support the findings of this study are available from the corresponding author upon reasonable request.

## Introduction

**Fig. 1.**
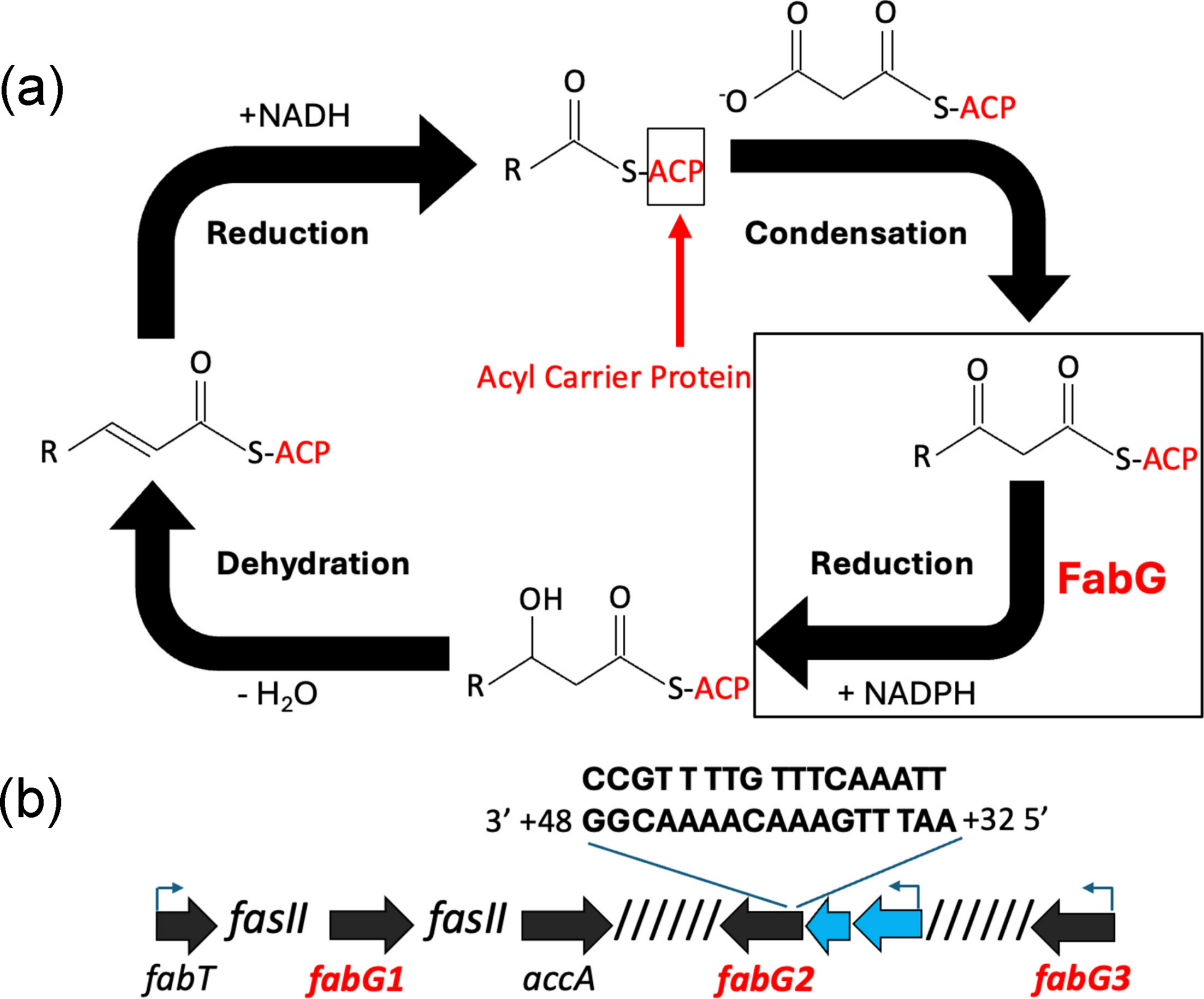
(A) Fatty acid biosynthesis in bacteria. (B) The location of the three *fabG* candidates on the *E. faecalis* genome. The boxed reaction is that catalysed by β-ketoacyl-ACP reductase (FabG). In panel (B), the three *E. faecalis fab* gene candidates are labelled in red, whereas the blue segments indicate the two genes of unknown function clustered with *fabG2*. The nucleotide sequence above *fabG2* is the putative FabT transcription factor-binding site of *fabG2* [16]. The genes between *fabT* and *fabG1* and those between *fabG1* and *accA* encode FASII proteins [27]. The slashes denote genomic distance. FASII, type II fatty acid synthesis.

Fatty acid synthesis provides precursors for phospholipid synthesis, secondary metabolite production, signal molecule formation and protein post-translational modification [1,3]. The pathway is the type II fatty acid synthesis system, a series of discrete enzymes found in bacteria, mitochondria and plant plastids [1]. In the paradigm *Escherichia coli*, and in other organisms, fatty acid synthesis proceeds through a cycle of four successive steps in which the intermediates are linked to an acyl carrier protein (ACP) by a thioester bond (Fig. 1a) [4]. This process is initiated by the condensation of malonyl-ACP with acetyl-CoA (or acyl-ACP) [4]. The β-ketoacyl-ACP intermediate produced by the condensation reaction is first reduced to a β-hydroxyl-acyl-ACP and then dehydrated to an enoyl-ACP, followed by a final reduction to give a saturated acyl-ACP elongated by two carbon atoms [4]. This cycle is repeated until the acyl-ACPs achieve the chain lengths required for phospholipid synthesis. Shorter acyl chains are precursors of vitamins (biotin and lipoic acid), lipid A or various signalling molecules [1,3,5].

**Fig. 2.**
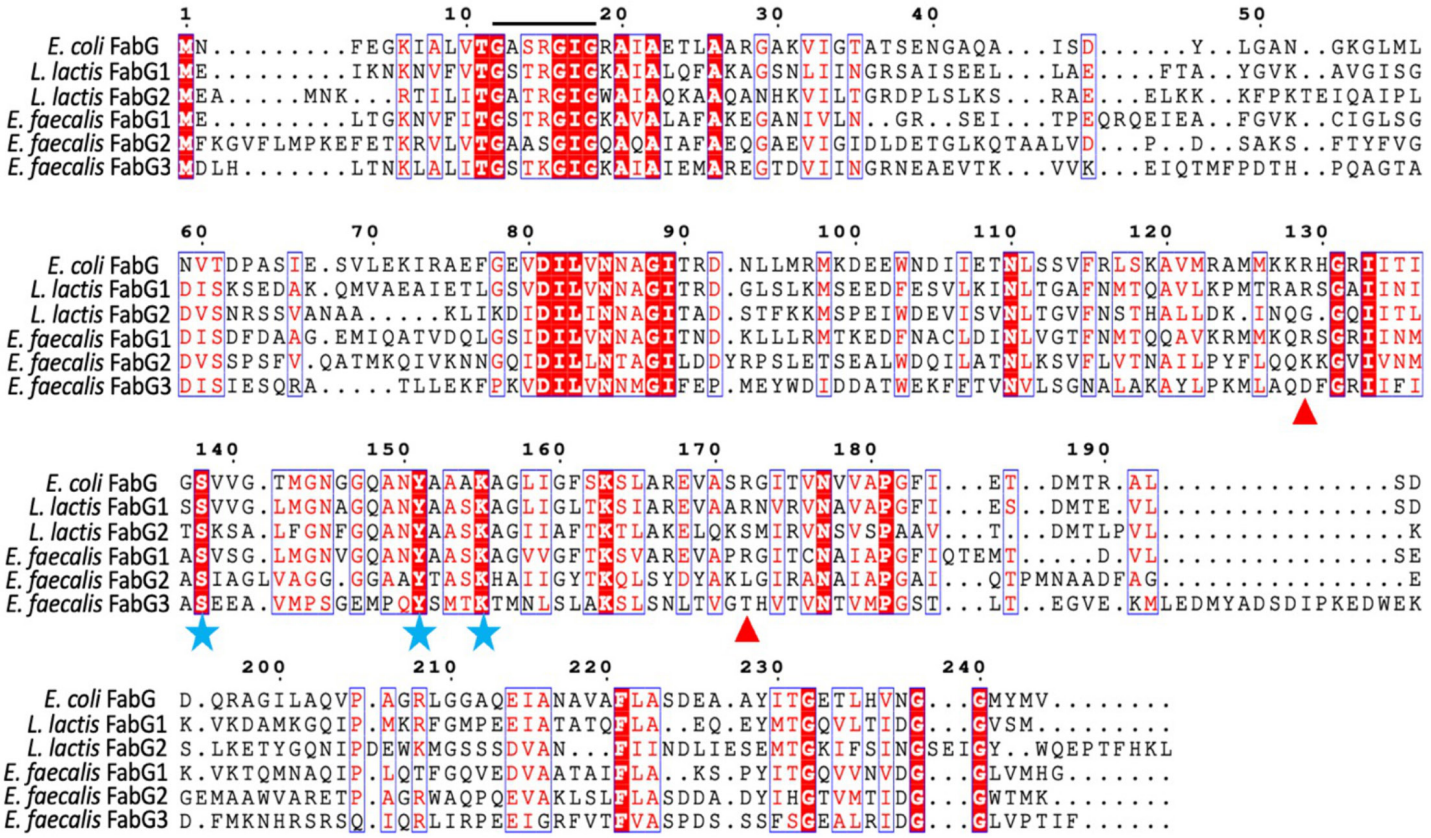
Alignments of *E. faecalis* FabG1, FabG2 and FabG3 with the β-ketoacyl-ACP reductases of *E. coli* and *L. lactis*. The cofactor-binding site (Gly-Xaa_3_-Gly-Xaa-Gly) is overlined, while the catalytic triad residue (Ser–Lys–Tyr) is marked with blue stars. The site marked with red triangles indicates the two arginine residues of *E. coli* FabG essential for ACP binding [29]. The red-shaded boxed residues denote strictly conserved residues, whereas the blue-boxed, red-coloured residues are positives as defined by the CLUSTALW program. The alignment was processed using the T-Coffee Multiple Sequence Alignment Server. The *L. lactis* FabG2 protein was previously shown to lack β-ketoacyl-ACP reductase activity, whereas the FabG1 of this bacterium had activity both *in vivo* and *in vitro* [25].The UniProt numbers are as follows: *E. coli*, P0AEK2; *L. lactis* FabG1, Q9CHF7; *L. lactis* FabG2, Q9CEQ0; *E. faecalis* FabG1, Q820T3; *E. faecalis* FabG2, Q833Z8 and *E. faecalis* FabG3, Q834I5.

β-Ketoacyl-ACP reductase (FabG), a member of the short-chain alcohol dehydrogenase/reductase (SDR) family, uses NADPH to catalyse the first of the two reductive steps of the fatty acid synthesis cycle (Fig. 1a) [8]. The FabG enzyme is a tetramer and contains a Ser–Lys–Tyr catalytic triad with an N-terminal cofactor-binding site (glycine motif) that is highly conserved (Fig. 2) [8,11]. FabG proteins can also be specialized β-ketoacyl-ACP reductases involved in synthesizing signalling molecules [8,9, 12].

*Enterococcus faecalis* is a commensal Gram-positive bacterium that inhabits the gastrointestinal tract of humans and often leads to difficult hospital-acquired infections in surgical patients due to its resistance to multiple commonly used antibacterial agents [13,15]. Three *fabG* candidates were identified through sequence alignment with *E. coli* FabG (UniProt P0AEK2) using the Kyoto Encyclopedia of Genes and Genomes (KEGG). The *E. faecalis fabG1* gene (UniProt Q820T3) is centrally located within the 12-gene *fab* operon that encodes most of the fatty acid synthesis enzymes (Fig. 1b) [16,18]. The putative *E. faecalis fabG2* gene (UniProt Q833Z8) is clustered with two genes encoding unknown proteins, whereas the putative * E. faecalis fabG3* gene (UniProt Q834I5) is unaccompanied (Fig. 1b). A putative binding site for the transcription factor FabT that regulates *E. faecalis* fatty acid synthesis was reported at the 5′ end of the *fabG2* coding region (Fig. 1b) [16].

We report the failure to detect β-ketoacyl-ACP reductase function for the putative *E. faecalis* FabG2 or FabG3 candidates by complementation of an *E. coli fabG*(Ts) strain and an *E. faecalis ∆fabG1* strain. We also report that the expression of *fabG2* is not regulated by FabT, in conflict with an earlier report [16].

## Methods

### Materials

All fatty acids and antibiotics were purchased from Sigma-Aldrich. The media were purchased from Fisher Scientific. The DNA polymerase, restriction endonuclease, T4 ligase and Gibson Assembly Cloning Kit were purchased from New England Biolabs. M17 Broth was purchased from Becton Dickinson. All other reagents were of the highest available quality. Oligonucleotide primers were synthesized by Integrated DNA Technologies, and DNA sequencing was performed by ACGT, Inc.

### Bacterial strains, plasmids and growth conditions

The bacterial strains and plasmids used in this study are listed in Table SI, available in the online Supplementary Material, and the primers used for this study are listed in Table SII. *E. coli* cultures were incubated at 37 °C in Rich Broth (RB) medium (tryptone, 10 g l^–1^; yeast extract, 1 g l^–1^; NaCl, 5 g l^–1^), whereas *E. faecalis* cultures were grown at 37 °C in M17 medium (BD DIFCO). Antibiotics were added at the following concentrations (in mg l^–1^): sodium ampicillin, 100 for *E. coli*; chloramphenicol, 30 for *E. coli*; gentamicin, 30 for *E. coli* and erythromycin, 250 for *E. coli* and 5 for *E. faecalis*. Hexanoic acid, octanoic acid, decanoic acid and oleic acid were added at 0.1 mM, while the *cis*-5-tetradecenoic acid was added at 0.01 mM. Arabinose was supplied at 0.02% (w/v).

### Construction of the *E. faecalis ∆fabG1* strain

Strain constructions were performed as described previously [19]. To construct the *∆fabG1* strain, the DNA cassette for a null ∆*fabG1* gene was composed of a 500 bp upstream segment (arm I) and a 500 bp downstream segment (arm II) of the *fabG1* gene coding region. The deletion cassette was constructed by overlap PCR using primer sets EffabG1 Arm 1 F and EffabG1 Arm 1 R, and EffabG1 Arm 2 F and EffabG1 Arm 2 R, and then ligated with the linearized temperature-sensitive * E. coli lacZ* gene vector pBVGh, amplified by primer set pBVGh F and pBVGh R, through Gibson assembly assay. *E. faecalis* cells transformed with the constructed plasmid above were selected on AC agar plates with 5 mg l^−1^ erythromycin and 100 mg l^−1^ 5-bromo-4-chloro-3-indolyl-β-d-galactopyranoside (X-Gal) at 30 °C. One blue colony was picked and streaked on AC agar plates with the same components at 42 °C to verify plasmid integration into the genome. A blue colony was cultured in AC liquid medium with 0.1 mM oleate at 30 °C for 4 h and then shifted to 42 °C overnight. This process was repeated several times, and the culture was diluted and plated on AC agar plates containing 0.1 mM oleate and X-Gal at 42 °C, and plated again on X-Gal plates containing 0.1 mM oleate. White colonies were selected and validated for deletion of the target gene by colony PCR.

### Construction of the *E. faecalis fabG1-*, *fabG2-* and *fabG3*-expression plasmids

The construction of *E. faecalis fabG*-expression plasmids was modified from the assay described previously [18,20, 21]. For the *fabG1*-expression plasmid, the *fabG1* gene was amplified from *E. faecalis* FA2-2 strain genomic DNA using primer set EffabG1 F and EffabG1 R, and the product was ligated with linearized pQZ28 vector amplified using primer set pQZ28 F2 and pQZ28-p32 R2 by Gibson assembly assay.

To construct the *E. faecalis fabG2*- or *fabG3*-expression plasmids, the *fabG2* and *fabG3* coding regions were amplified from genomic DNA using primer sets EffabG2 *SmaI* F and EffabG2 *EcoRI* R, and EffabG3 *SmaI* F and EffabG3 *EcoRI* R, respectively, and then inserted into the *NcoI* and *EcoRI* sites of vector pQZ28 [20,22].

### Functional identification of *E. faecalis* FabG1, FabG2 and FabG3

To express *E. faecalis* FabG candidates in *E. coli*, the coding regions of *E. faecalis fabG1*, *fabG2* and *fabG3* were amplified from genomic DNA by primer sets EffabG1 *NdeI* F and EffabG1 *PstI* R, EffabG2 *NdeI* F and EffabG2 *PstI* R, and EffabG3 *NdeI* F and EffabG3 *PstI* R, respectively, and then inserted into the *NdeI* and *PstI* sites of the pBAD24M vector downstream of the arabinose promoter [22]. The *E. faecalis fabG*-expression plasmids were transformed into *E. coli fabG*(Ts) strain CL104, and the transformants were tested for growth on RB agar medium at 37 °C [23].

### β-Galactosidase assays

The *lacZ* reporter plasmids expressing *E. coli* β-galactosidase from the promoters of *E. faecalis fabG2* and *fabG3* were constructed as described previously [18]. The promoter sequence, together with the first 35 bp of the coding sequence (−1448 to −914 relative to the *fabG2* gene initiation codon ATG or −500 to +35 relative to the *fabG3* gene initiation codon ATG), was amplified by primer set P-EffabG2C plus 35 *PstI* F and P-EffabG2C plus 35 *SalI* R or P-EffabG3 plus 35 *PstI* F and P-EffabG3 plus 35 *SalI* R, and then inserted into the *PstI* and *SalI* sites at the 5′ end of the promoterless *lacZ* gene of plasmid pBHK322 constructed as in the previous work [24].

β-Galactosidase activity was assayed as previously described [18,22, 24]. Briefly, *E. faecalis* strains transformed with the *lacZ* reporter plasmids above were cultured to mid-log phase at 37 °C, and the harvested cells were washed by PBS, resuspended in Z buffer, lysed with SDS and chloroform and assayed for β-galactosidase activity. The data were collected in triplicate.

## Results

### Complementation of the *E. coli fabG*(Ts) strain with *E. faecalis fabG* genes

Comparison of the amino acid sequences of the FabG proteins of several species showed 58% identity between *E. faecalis* FabG1 and *Lactococcus lactis* FabG1 (Fig. 2), the functional β-ketoacyl-ACP reductase of that bacterium [25]. However, although the bacteria are close relatives, *E. faecalis* FabG2 or FabG3 had only ~30% identity to *L. lactis* FabG1 (Fig. 2). The *E. faecalis* FabG1, FabG2 and FabG3 had ~44%, 36% and 29% identity, respectively, to the *E. coli* FabG β-ketoacyl-ACP reductase (Fig. 2). Among the three *E. faecalis* proteins, the identities were G1–G2, 34%; G1–G3, 27% and G2–G3, 25%.

To test the function of *E. faecalis* FabG2 and FabG3 in fatty acid synthesis, the *fabG1*, the putative *fabG2* and the *fabG3* genes were inserted into the pBAD24M vector, and the resulting plasmids were transformed into the *E. coli fabG*(Ts) strain CL104. The strain CL104 *fabG* gene encodes an E233K/A154T FabG protein [23]. The tetrameric FabG is essentially a dimer of dimers and thus has two types of subunit interfaces. The E233K mutation alters the interface between dimers that assemble the tetramer, whereas the A154T mutation lies at the interface for dimer formation. The separated mutations had diverse effects. The E233K protein is very thermolabile, whereas the A154T mutation lacked a phenotype, although it potentiated the thermolability of the E233K enzyme [23].

**Fig. 3.**
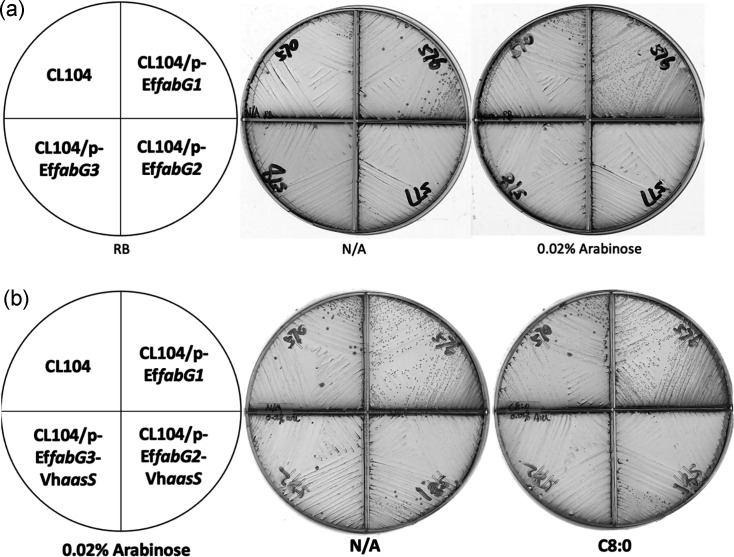
Complementation of the *E. coli fabG*(Ts) strain CL104 with *E. faecalis fabG* candidates. (A) Growth of *E. coli* CL104 strain expressing *E. faecalis fabG1*, *fabG2* or *fabG3,* respectively, on RB agar medium. (B) Growth of *E. coli* CL104 strain co-expressing either *E. faecalis fabG2* or *fabG3* and *V. harveyi aasS* on RB agar medium supplied with octanoic acid (C8 : 0). The bacteria were cultured at 37 °C in both panels. *V. harveyi aasS* encodes acyl-ACP synthetase [32]. AasS, acyl-ACP synthetase; N/A, no additions.

The transformants were plated at 37 °C on RB agar medium in the presence of arabinose [23]. The expression of the *E. faecalis fabG* genes was under control of the arabinose-regulated *araBAD* promoter. Expression of *E. faecalis* FabG1 allowed growth of strain CL104 (Fig. 3a), demonstrating β-ketoacyl-ACP reductase activity. However, neither FabG2 nor FabG3 restored growth under these conditions (Fig. 3a). These data indicated that FabG2 and FabG3 could not replace FabG function in *E. coli*. In addition, no growth was seen when *Vibrio harveyi* acyl-ACP synthetase was co-expressed in the presence of octanoic acid (C8 : 0) or hexanoic acid (C6 : 0) (Fig. 3b and data not shown), in attempts to bypass β-ketoacyl-ACP reductase function in the early steps of the pathway. This supplementation was successful in *Xanthomonas* [9], but not in *E. faecalis*. These data argue that FabG1 is the only functional *E. faecalis* β-ketoacyl-ACP reductase.

### Complementation of the *E. faecalis ∆fabG1* strain with *E. faecalis fabG* genes

**Fig. 4.**
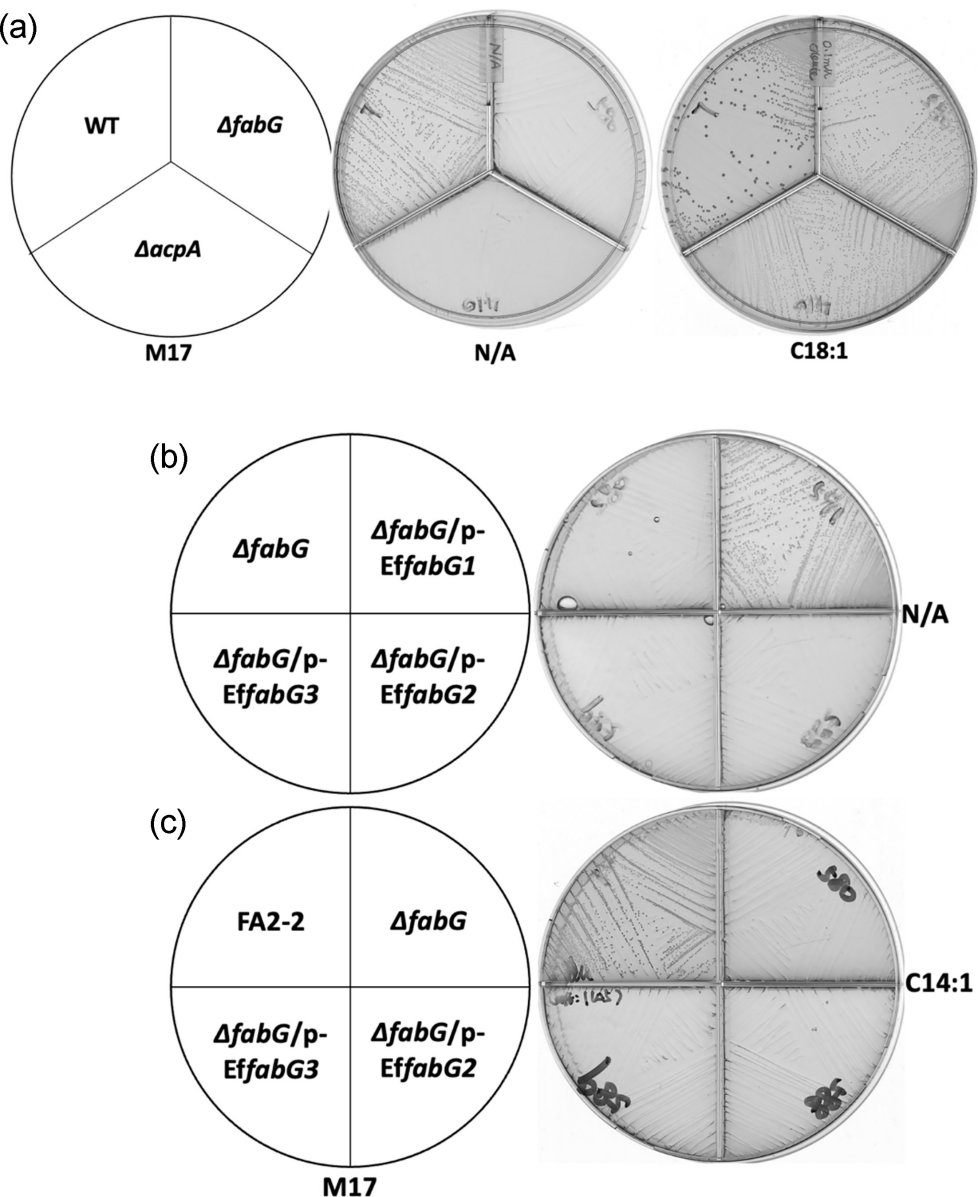
Complementation of *E. faecalis ∆fabG1* strain with *E. faecalis fabG* candidates. (A) Growth of the *E. faecalis ∆fabG1* strain on M17 agarose medium with or without supplementation with exogenous oleate (C18 : 1). (B) Growth of the *E. faecalis ∆fabG1* strain overexpressing FabG1, FabG2 or FabG3 on M17 agarose medium. (C) Growth of the *E. faecalis ∆fabG1* strain overexpressing FabG2 or FabG3 on M17 agarose medium supplied with *cis*-5-tetradecenoic acid (C14 : 1). FA2-2 is the *E. faecalis* WT strain. N/A, no additions; WT, wild-type.

The failure to restore growth of the *E. coli* CL104 strain by expression of *E. faecalis* FabG2 or FabG3 might be due to the known structural [26]and functional [26] differences between the different ACPs of these two species. To test complementation in the cognate bacterium, we constructed a strain lacking *fabG1*. We found the *∆fabG1* strain to be a fatty acid auxotroph, as seen for other *E. faecalis ∆fab* strains [22,27], indicating that FabG2 and FabG3 cannot replace FabG1. Deletion of *fabG1* blocked growth of *E. faecalis,* which was relieved either by supplementation with oleic acid or by complementation with *E. faecalis fabG1* (Fig. 4a, b). Moreover, plasmids encoding *fabG2* or *fabG3* failed to support growth of the *E. faecalis ∆fabG* strain even when *cis*-5-tetradecenoic acid (*cis*-5 C14) was supplied (Fig. 4c), a supplement that can bypass the initiation step of fatty acid synthesis [28]. These data demonstrated that FabG1, but not FabG2 or FabG3, functioned as the β-ketoacyl-ACP reductase of *E. faecalis* fatty acid synthesis.

### Expression of the *E. faecalis fabG2* and *fabG3* genes

**Fig. 5.**
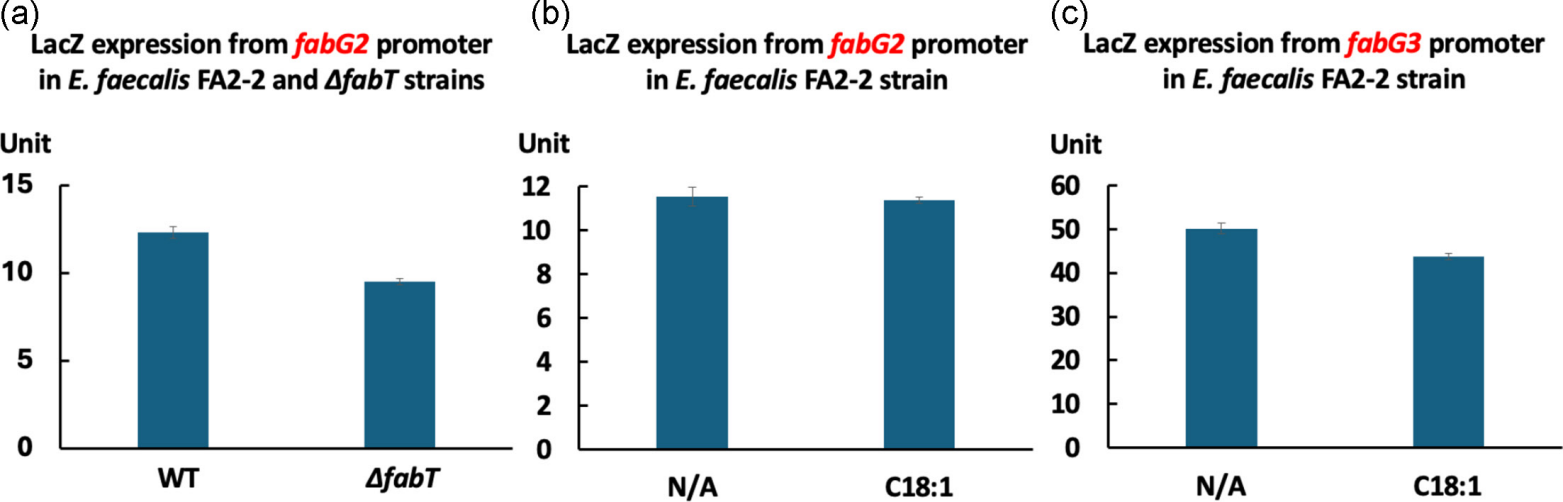
β-Galactosidase assay for expression of *E. faecalis fabG2* or *fabG3* gene *in vivo*. (A) β-Galactosidase expression from *fabG2* promoter in *E. faecalis* WT (FA2-2) and *∆fabT* strains. β-Galactosidase expression from the *fabG2* (B) or *fabG3* (C) promoters in the *E. faecalis* WT strain with or without oleic acid (C18 : 1) supplementation. Note that β-galactosidase expression from the *fabG2* promoter is slightly greater than that of a fusion to the weak constitutive promoter of the thioesterase-encoding *tesE* gene which is not controlled by FabT [4] (Fig. S1). Therefore, *fabG2* seems a weakly expressed gene like *tesE*. The background in the WT strain is barely detectable, perhaps 0.2–0.5 units. N/A, no additions; WT, wild-type.

A previous study reported that *E. faecalis fabG1* was co-transcribed with the genes of the *fab* operon under regulation by the FabT transcription factor [16]. Moreover, a possible FabT-binding site (+32 to +48 relative to the *fabG2* gene initiation codon ATG) was identified for the *fabG2* gene (Fig. 1b) [16]. To further explore the expression of the *fabG2* and *fabG3* genes *in vivo*, the β-galactosidase reporter plasmids were constructed by fusing the promoters of *fabG2* or *fabG3* to the promoterless *E. coli lacZ* gene of plasmid pBHK322 [18,24]. The constructed plasmids were then transformed into *E. faecalis* wild-type or *∆fabT* strains for β-galactosidase assays. Unlike the genes of the *fab* regulon, where FabT acts as a repressor [16,18], deletion of *fabT* resulted in only a modest 20% reduction in β-galactosidase expression driven by the *fabG2* promoter relative to the wild-type strain (Fig. 5a). Furthermore, exogenous oleic acid, which potentates FabT repression, failed to decrease β-galactosidase expression from either the *fabG2* or *fabG3* promoters in the wild-type strain (Fig. 5b, c). These data indicate that the expression of the *fabG2* and *fabG3* candidates is not regulated by FabT. Note that no recognizable promoter could be identified in the 500 bp segment upstream of the *E. faecalis fabG2* gene (−500 to −1 relative to the initiation codon ATG; data not shown).

## Discussion

This work tested the function and expression of putative FabG candidates encoded by *E. faecalis*. Only FabG1 had β-ketoacyl-ACP reductase activity and supported *de novo* fatty acid synthesis in *fabG* strains of *E. coli* and *E. faecalis*. In both bacteria, the putative FabG2 and FabG3 proteins failed to replace the long-chain β-ketoacyl-ACP substrate *in vivo* (Figs3  4). Moreover, the expression of the *fabG2* and *fabG3* genes was not repressed by the FabT transcription factor or by supplementation with oleic acid (Fig. 5).

The inability of FabG2 and FabG3 to provide β-ketoacyl-ACP reductase activity *in vivo* (Figs3  4) could be due to an inability to interact with ACP, or that the proteins function in other pathways, as discussed below. Two *E. coli* FabG arginine residues (R129 and R172), reported to be essential in binding ACP [29], are lacking in both *E. faecalis* FabG2 and FabG3 but are present in *E. faecalis* FabG1 (Fig. 2). Although *Xanthomonas campestris* pv. *campestris* FabG2 is active despite lacking these residues [9], no detectable activity was observed in the *E. faecalis* FabG2 or FabG3 candidates (Figs3b  4c). Note that *E. faecalis* AcpA differs from canonical bacterial ACPs in that it lacks the conserved helix 3 [30].

The expression of the *E. faecalis fabG2* gene was significantly weaker than the expression of the *fabG1* and *fabG3* genes (Fig. 5b, c). Although previous work reported a significant decrease in *fabG2* transcription in the presence of human serum [16], we observed only marginal effects on β-galactosidase expression from the *fabG2* promoter either by deletion of *fabT* or by supplementation with oleic acid (Fig. 5a, b). Human serum is replete with long-chain fatty acids [31], but there are many other serum components that could account for the reported repression of *fabG2* [16]. Indeed, *fabG2* has been annotated as a glucose/ribitol dehydrogenase in InterPro (IPR002347).

These annotations illustrate the difficulty in assigning FabG genes [25]. These enzymes are members of a very large protein family, the SDR family, which use NADH or NADPH as the reductant. Many SDR enzymes are involved in monosaccharide synthesis pathways (e.g. gluconate 5-dehydrogenase). However, there is an SDR that catalyses a reaction similar to FabG, the β-hydroxyacyl-CoA dehydrogenase of the β-oxidation pathway of fatty acid catabolism. β-Hydroxyacyl-CoA is not a plausible FabG2 or FabG3 candidate because *E. faecalis* lacks β-oxidation pathway genes and does not shorten exogenous fatty acids [18]. Our data indicate that the FabG1 designation should be dropped since *E. faecalis* has only a single FabG.

## Supplementary material

10.1099/mic.0.001610 Uncited Supplementary Material 1.

## Abbreviations

ACP: acyl carrier protein
KEGG: Kyoto Encyclopedia of Genes and Genomes
RB: Rich Broth
SDR: short-chain alcohol dehydrogenase/reductase
