# Supplementary material for: Two putative Enterococcus faecalis fabG genes do not encode β-ketoacyl-acyl carrier protein reductases
Source: Microbiology (Reading). 2025 Sep 24;171(9):001610. doi: 10.1099/mic.0.001610 (PMC13293336; doi:10.1099/mic.0.001610)
Supplement: Uncited Supplementary Material 1. [file mic-171-01610-s001.pdf]

Supplement to

Homologues encoded by *Enterococcus faecalis* two redundant *fabG* genes are not β-Ketoacyl-Acyl Carrier Protein Reductase

Qi Zou, Huijuan Dong and John E. Cronan

Table SI Strains and Plasmids

Strains and Plasmids	Description	Source
**Strains**		
E. faecalis FA2-2	Wild Type	Lab Store
E. faecalis ZL116	Δ*fabT*	[17]
E. faecalis DHJ518	Δ*fabG1*	This work
E. faecalis QZ591	Δ*fabG1* with *fabG1* on pQZ28 plasmid	This work
E. faecalis QZ588	Δ*fabG1* with *fabG2* on pQZ28 plasmid	This work
E. faecalis QZ589	Δ*fabG1* with *fabG3* on pQZ28 plasmid	This work
E. faecalis DHJ484	Δ*acpA*	[28]
E. faecalis QZ556	FA2-2 with *lacZ* expression plasmid from *fabG2* promoter	This work
E. faecalis QZ557	FA2-2 with *lacZ* expression plasmid from *fabG3* promoter	This work
E. faecalis QZ564	Δ*fabT* with *lacZ* expression plasmid from *fabG2* promoter	This work
E. coli CL104	*fabG*(TS) *panD*, Cm^r, Tet^r, Km^r	[23]
E. coli QZ576	CL104 with *fabG1* on pBAD24M plasmid	This work
E. coli QZ577	CL104 with *fabG2* on pBAD24M plasmid	This work
E. coli QZ578	CL104 with *fabG3* on pBAD24M plasmid	This work
E. coli QZ581	CL104 with *fabG2* on pBAD24M plasmid and *aasS* on pBAD33-Gm plasmid	This work
**Plasmid**		
pQZ28	Shuttled plasmid vector with a p32 promoter modified from pZL277 by replacing the chloramphenicol-resistant gene with erythromycin-resistant gene, *E. faecalis* expression	[21]

pBAD24M	Expression vector modified from pBAD24 by replacing *NcoI* site with *NdeI* site, *E. coli* expression	[22]
pBHK322	promoterless *E. coli* lacZ on vector pTRKL2	[24]
pBVGh	Temperature-sensitive β-galactosidase erythromycin-resistant gene modification vector	[19]
pDHJ517	*E. faecalis fabG1* knockout cassette on vector pBVGh	This work
pQZ573	*E. faecalis fabG1* in pBAD24M	This work
pQZ574	*E. faecalis fabG2* in pBAD24M	This work
pQZ575	*E. faecalis fabG3* in pBAD24M	This work
pQZ552	*E. faecalis fabG2* promoter region (-1448 to -914) at 5'-end of *lacZ* in pBHK322	This work
pQZ555	*E. faecalis fabG2* promoter region (-500 to +35) at 5'-end of *lacZ* in pBHK322	This work
pDHJ513	*E. faecalis fabG1* in pQZ28	This work
pQZ584	*E. faecalis fabG2* in pQZ28	This work
pQZ586	*E. faecalis fabG3* in pQZ28	This work
pHZ50	*V. harveyi aasS* in pBAD33-Gm^r	[9]

Table SII Oligonucleotides primers used in the study

**Primers***	**Sequence 5'-3'**
pQZ28 F2	GAATTCGTAATCATGTCATAGCTGTTT
pQZ28-p32 R2	GGTTCACCTCCTTTTATTTTTTTACCTAC
EffabG1 F	GTAGGTAAAAAAATAAAAGGAGGTGAACCATGGAATTAACAGGAAAAAACGT
EffabG1 R	AAACAGCTATGACATGATTACGAATTCTTATCCGTGCATGACTAAGC
EffabG2 SmaI F	TCCCCCGGGCTATGTTTAAAGGAGTTTTTCTTATGC
EffabG2 EcoRI R	CCGGAATTCTTATTTCATGGTCCAACCA
EffabG3 SmaI F	TCCCCCGGGCTATGGATTTACACTTAACGAATAAATTA
EffabG3 EcoRI R	CCGGAATTCTTAGAAGATCGTTGGAACTAA
P-EffabG2C plus 35 PstI F	AAAATTCTGCAGTTAAAAATATTATAGTGTAAAGTTAATTTTTGT
P-EffabG2C plus 35 SalI R	ACGCGTCGACCCATCTTGTTTGACTGTAATAT
P-EffabG3 plus 35 PstI F	AAAATTCTGCAGCAATCACAGTAATAATTTCTTCTTCT
P-EffabG3 plus 35 SalI R	ACGCGTCGACATTAATGCTAATTTATTCGTTAAGTGT
EffabG1 NdeI F	GGGAATTCCATATGGAATTAACAGGAAAAAACGT

EffabG1 PstI R	AAAATTCTGCAGTTATCCGTGCATGACTAA
EffabG2 NdeI F	GGGAATTCCATATGTTTAAAGGAGTTTTTCTTATGC
EffabG2 PstI R	AAAATTCTGCAGTTATTTCATGGTCCAACCA
EffabG3 NdeI F	GGGAATTCCATATGGATTTACACTTAACGAATAAA T
EffabG3 PstI R	AAAATTCTGCAGTTAGAAGATCGTTGGAACTA
EffabG1 Arm 1 F	ATCGATGCATGCCATGGTACCCGGGAGCTCAAGAA GCCAGTGCTTTCGGAA
EffabG1 Arm 1 R	AATATTGGCGCCCTCTTTCG
EffabG1 Arm 2 F	CGAAAGAGGGCGCCAATATTACTGGGCAAGTCGT GAATGT
EffabG1 Arm 2 R	TAGAACTAGTAGGGATCCCCCGGGCTGCAGTCCTT TGGCACCAACACGTA
pBVGh F	CTGCAGCCCGGGGGAT
pBVGh R	GAGCTCCCGGGTACCATG

* The primer sequences were based on the *E. faecalis* V583 genome. The underlined sequences indicate the restriction sites used in the study.

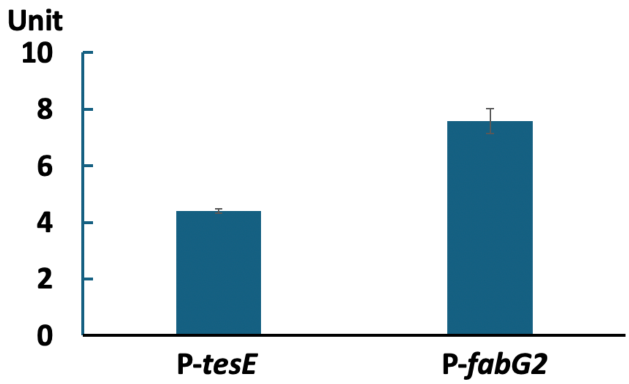

Fig S1. Comparison of β-galactosidase expression of fusions to the *tesE* and f*abG2* promoters. The *tesE* promoter is not subject to FabT repression [21].
